# Nitric oxide-releasing responsive biomaterials for antimicrobial skin therapy

**DOI:** 10.1016/j.redox.2026.104182

**Published:** 2026-04-23

**Authors:** Kelli C. Freitas Mariano, Joana C. Pieretti, Renan S. Nunes, Roberta A. dos Reis, Victor D.P. Cinel, Cecília B. Aragão, Morgana Halfeld, Tanveer A. Tabish, Amedea B. Seabra

**Affiliations:** a Center for Natural and Human Sciences, Federal University of ABC (UFABC), Santo André, SP, 09210-580, Brazil; b Division of Cardiovascular Medicine, Radcliffe Department of Medicine, British Heart Foundation (BHF) Centre of Research Excellence, University of Oxford, Oxford, OX3 7BN, United Kingdom

**Keywords:** Nitric oxide, Responsive, Biomaterials, Antimicrobial, Skin

## Abstract

Nitric oxide (NO) is a key signaling molecule involved in many physiological processes including vasodilation, neurotransmission, inflammatory and antimicrobial responses. Despite its therapeutic potential, its clinical utility remains limited due to its extremely short half-life (less than 5 s), high reactivity (with oxygen radicals) and the need for precise spatiotemporal dose control. To overcome these limitations, recent advancements in biomaterials have led to the development of responsive systems capable of delivering NO in a targeted and controlled manner. These systems can respond to endogenous stimuli (such as pH changes, enzymatic activity, altered redox environment (associated with hypoxia, oxidative stress) which are key characteristics of diseased cells) or to exogenous stimuli (such as light, X-ray, ultrasound). By allowing site-specific and time-controlled NO release, these responsive biomaterials offer improved control over local NO delivery and are being evaluated predominantly in *in vitro* and animal models. In this review, we discuss the advances in design of NO-releasing responsive biomaterials with a particular focus on applications in antimicrobial skin therapy. This review bridges materials bioengineering and dermatology to explore the promise of NO-based therapies for tackling skin and wound care problems.

## 1 Introduction

Skin injuries resulting from accidents, trauma, surgery or chronic disease, affect hundreds of millions worldwide each year and continue to represent a major clinical socioeconomic burden [1]. In diabetic foot ulcers, only a small proportion heal effectively, many lead to chronic non-healing wounds and around 20% progress to amputation [2]. Venous leg ulcers, affecting 1–3% of the population with higher rates in older age groups are associated with persistent inflammation and defective angiogenesis, resulting in chronic wounds that fail to heal [3]. Pressure ulcers occur in up to 12.8% of hospitalized patients [4] further indicating that hard-to-heal wounds are clinically severe. Chronic wounds and biofilm-related skin infections affect over 50 million people worldwide and the rise of antimicrobial resistance (AMR) is predicted to cause 10 million deaths annually by 2050 [5]. Traditional interventions for the treatment of chronic wounds and infections typically include topical antibiotics and dressings, which often fail against persistent infections and biofilms [6]. Antibiotics can target individually living pathogens and strains, but they have limited efficacy against biofilms which are formed by diverse microbial species from multiple kingdoms. Next generation nitric oxide (NO)-based treatments are promising, as NO can improve skin regeneration, combat resistant pathogens and help preserve the natural skin microbiota [7]. Notably, the majority of NO-releasing biomaterial studies in dermatology remain at the *in vitro* or small animal stage, with relatively limited clinical evidence to date. NO plays an important role in wound healing, infection control, tissue regeneration and dermal vasodilation [[8], [9], [10]]. NO-donating drugs exemplified by glyceryl trinitrate (GTN) have been used to treat angina for over 140 years, but they release non-specific doses of NO in the circulation. Furthermore, the broad therapeutic spectrum of NO is hindered by challenges such as its short half-life (less than 5 s), rapid reactivity (with oxygen radicals, heme) and the challenge of achieving precise dose control [11]. For the development of efficient NO-based therapeutic strategies, it is essential to deliver the right amount of NO at the right place and time [12]. To overcome these limitations, targeted NO delivery systems have been devised and developed, for example using biomaterials, which facilitate more targeted, effective and sustained release of NO at the site of action. Very recently, responsive biomaterials capable of delivering NO in response to endogenous stimuli, such as pH changes, enzymatic activity, redox potential (hypoxia, oxidative stress) or temperature variations have received increasing attention. By allowing site-specific and time-controlled NO release, these platforms could improve therapeutic precision, improve efficacy while minimize side effects to non-diseased surrounding healthy cells and tissues [13,14].

Although NO-releasing nanomaterials have been extensively reviewed in other contexts, including cardiovascular applications [15], and anticancer applications [16], their potential in antimicrobial skin therapy remains comparatively underexplored. For example, recently, Friedman and co-workers reported a review on the development of nanomaterials in dermatology, focusing on the treatment of skin and related tissue infections [17]. However, key challenges and the specific application of responsive NO delivery systems in skin-targeted therapies remain insufficiently addressed. To address this gap, our review discusses the latest advancements in stimuli-responsive hydrogels, nanocarriers and polymeric scaffolds specifically tuned for skin applications. We discuss the design, mechanisms of action and therapeutic advantages of these responsive systems as well as the optimized NO delivery strategies. Additionally, we also discuss the challenges for their translational use, focusing on the need to bridge the gap between materials bioengineering and dermatology.

## 2 Classes of biomaterials

Biomaterials are generally classified as natural or synthetic (engineered) based on their origin and composition [[18], [19], [20]]. Natural biomaterials are obtained from biological sources such as animals, plants, or microbial tissues. Typical examples are collagen, gelatin, chitosan, alginate, hyaluronic acid, silk fibroin and bacterial cellulose. These materials exhibit good biocompatibility and biodegradability and often mimic the extracellular matrix (ECM), which facilitates cell adhesion, migration and proliferation. For example, collagen is extensively used in skin substitutes due to its low immunogenicity and its ability to support tissue repair [21]. Whereas chitosan, a cationic polysaccharide derived from chitin, offers hemostatic, film-forming and intrinsic antimicrobial properties, making it particularly suitable for treating wounds [22,23]. Despite their promising biofunctionality and regenerative promise, natural biomaterials have limitations, including low mechanical strength and the risk of immunogenic responses, particularly when derived from animal sources. To address these limitations, chemical crosslinking or blending with synthetic polymers may improve structural stability.

Synthetic polymers such as polyethylene glycol (PEG) and biodegradable polyesters are widely used because they allow control over degradation rate, mechanical strength and drug release [23,24]. These materials can be chemically modified to carry NO donors or physically encapsulate them. For example, PEG is extensively used in wound dressings due to its good biocompatibility and hydrophilic nature, which supports moisture retention [25,26]. Although some synthetic polymers are biodegradable, their degradation can produce potentially toxic by-products such as lactic and glycolic acids, which may induce inflammatory responses. Whereas some synthetic polymers are biologically inert and therefore require strategies such as surface modification or the incorporation of bioactive motifs (e.g., peptides) to improve cell interactions [27]. Other synthetic materials including inorganic nanoparticles (e.g., silica, zinc oxide, ceria, gold and silver) [28] carbon-based nanomaterials (e.g., graphene oxide and carbon nanotubes) [29] and metal-organic frameworks (MOFs) [30] have been studied for skin antimicrobial therapies. Typically, silver nanoparticles have been used for their broad-spectrum antimicrobial actions, whereas graphene-based materials have been studied for both antibacterial activity and support for tissue regenration owing to their high specific surface area and ease of functionalization with different biomolecules and polymers [31].The combination of these synthetic (nano)materials with polymers as multifunctional composite platforms may facilitate high drug loading and controlled release for wound healing as well as tissue regeneration.

To address the individual limitations of natural and synthetic materials, hybrid composite materials that combine both materials classes have gained huge attention. Hybrid materials combine the biological performance of natural polymers with the mechanical strength and structural stability of synthetic materials [32,33]. Together, these materials form the basis for engineering NO-releasing systems for skin applications.

## 3 NO in skin health

### 3.1 Role of endogenous NO in skin

The first evidence of NO synthesis in human skin was reported more than 30 years ago [34]. It is now clinically established that NO plays a crucial role in maintaining skin health. Low production of NO causes several pathologies such as cardiovascular diseases, infections and delayed wound healing. In biological systems, NO is produced by NO synthase (NOS), which exist in three different isoforms; (i) constitutive - neuronal (nNOS/NOS1) and endothelial (eNOS/NOS3) are constitutively expressed and calcium and calmodulin-dependent, producing NO at low (pico-to nanomolar) concentrations. nNOS is expressed in neurons and skeletal and cardiac muscle, where it regulates neurotransmission and contributes to vasodilation via neural control of vascular tone; (ii) eNOS, is expressed in endothelial cells, is the key mediator of endothelium-dependent vasodilation and vascular homeostasis and (iii) whereas, inducible (iNOS/NOS2) is calcium-independent and produces higher amounts of NO for extended periods, (micromolar to millimolar concentrations) during inflammatory or immune responses. Under physiological conditions, the skin naturally produces NO, which plays a role in immune defense, vasodilation and tissue repair. However, in cases of chronic wounds or severe infections, NO levels become dysregulated, leading to delayed healing and increased bacterial susceptibility [35]. In chronic wounds, prolonged inflammation results in elevated iNOS expression and sustained NO production, causing oxidative stress and delayed healing, whereas insufficient eNOS-derived NO limits angiogenesis and collagen deposition [36,37].

**Fig. 1:**
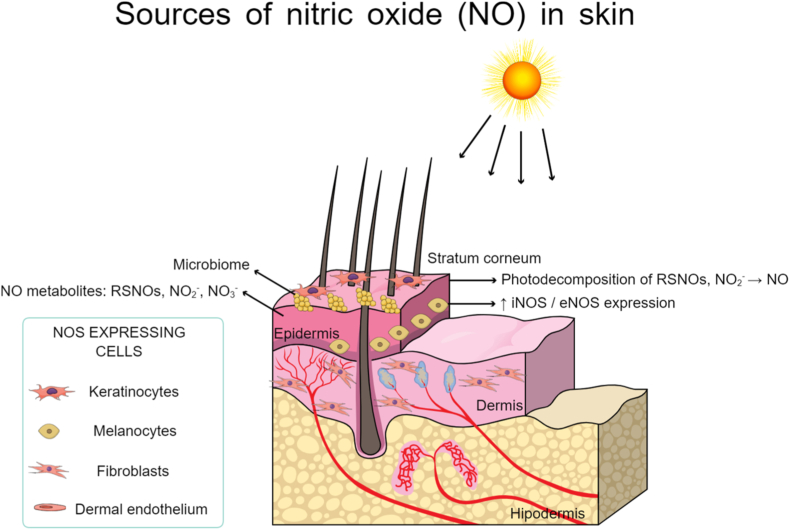
Schematic representation of the skin layers and major nitric oxide (NO) sources, including NOS isoforms in keratinocytes, fibroblasts, melanocytes and dermal microvasculature, as well as surface reservoirs of NO metabolites (nitrate, nitrite, *S*-nitrosothiols - RSNO). Ultraviolet (UV) light and the skin microbiome contribute to additional NO generation via photodecomposition and bacterial reduction of NO_3_^−^/NO_2_^−^ at the skin surface.

The skin is the largest organ of the human body, composed of two main layers: the epidermis and the dermis. Beneath these lies the hypodermis (or subcutaneous tissue), which anchors the skin to underlying structures [38]. Key cell types within these layers are endothelial cells, keratinocytes, fibroblasts and melanocytes, all capable of expressing NOS and producing NO, as represented in Fig. 1. All three isoforms of NOS are expressed in keratinocytes. NO production is primarily regulated by eNOS and nNOS [39]. nNOS (NOS1) primarily functions in neurotransmission and mediates nitrergic smooth muscle relaxation in tissues (such as gastrointestinal tract, airways, corpus cavernosum), contributing secondarily to vasodilation via perivascular nerves. eNOS (NOS3) is the major source of endothelium-derived NO that controls basal vasodilation and blood pressure and promotes angiogenesis and inhibits platelet aggregation and leukocyte adhesion. Under pathophysiological conditions such as inflammation, infection and chronic wounds, NO synthesis is upregulated via iNOS, exerting either protective or detrimental effects depending on its concentration and duration of exposure and the local redox environment (such as concomitant superoxide can drive peroxynitrite formation, inducing tissue damage) [40]. In keratinocytes and other epidermal cells, NO production is modulated by exogenous factors such as mechanical stimulation of the skin, disruption of the permeability barrier and ultraviolet (UV) exposure, mediated in part by nNOS and eNOS [39]. Beyond keratinocytes, other skin cells also contribute to NO production. Endothelial cells within the dermal microvasculature constitutively express eNOS under normal conditions but can be induced to express iNOS in response to pro-inflammatory cytokines or UVA stimulation. Similarly, melanocytes express eNOS and respond to UV irradiation by synthesizing melanin, which is stored in melanosomes [41] Notably, nNOS is also present in melanocytes, particularly in malignant melanomas [42]. In addition to the endogenous production of NO via NOS enzymes, the exogenous administration of NO has emerged as a promising strategy for achieving targeted therapeutic outcome. By modulating key physiological processes, exogenous NO delivery can improve wound healing, promote angiogenesis and regulate inflammatory responses, making it a valuable tool in regenerative medicine and dermatology.

Therapeutic NO delivery has been shown to stimulate angiogenesis, regulate inflammation and restore normal healing functions, making it a powerful therapeutic molecule in dermatology and regenerative medicine [43]. Topical application of NO-releasing formulations including gels and dressings, in animal and early human studies has been reported to accelerate wound closure, reduce microbial burden and improve local tissue oxygenation [44,45]. Despite its benefits, conventional NO donors such as *S-*nitrosothiols (RSNO such as *S*-nitrosoglutathione (GSNO)) and diazeniumdiolates exhibit condition-dependent and often rapid NO release (e.g., under acidic pH, light or metal ion catalysis) [12], leading to premature depletion and limiting their long-term therapeutic efficacy [46]. To overcome this, stimuli-responsive materials, nanoparticles and hydrogel-based delivery systems have been engineered to support site-specific, time-controlled NO release, optimizing its therapeutic action.

### 3.2 NO in skin wound healing

NO is involved in all phases of wound healing, *i.e*. inflammatory phase, proliferative phase and remodeling phase [36]. It affects inflammation, cell growth, collagen production, angiogenesis and tissue remodeling [47]. During the early inflammatory stage of wound healing, NO regulates inflammation by modulating the immune cells activity and influencing the cytokines release. The modulation of cytokine release in skin wounds reduces excessive neutrophil infiltration and promotes resolution of inflammation, thereby facilitating the re-epithelialization. During the proliferative stage, NO supports fibroblast migration and proliferation leading to improved dermal matrix formation and improved wound tensile strength [47].

In addition, NO is an important mediator of angiogenesis. It facilitates the development of new microvessels within the wound bed, which improves the local delivery of oxygen and nutrients. It also stimulates endothelial cell proliferation and migration, improving vascularization and overall blood supply to wounds [48]. NO also controls tissue remodeling by regulating matrix metalloproteinases (MMPs) and their inhibitors and prevents ECM accumulation and reduces the risk of hypertrophic scarring. [49]. For example, researchers examined the use of the RSNO as a NO donor in diabetic wound healing [50]. The results indicated that external NO supplementation may support the impaired healing related to diabetes, suggesting the potential of NO donors to improve tissue repair processes [50]. These findings demonstrate the therapeutic promise of NO in wound repair and tissue regeneration.

### 3.3 Therapeutic potential of exogenous NO

NO at higher concentrations (μM – mM) exhibits significant antimicrobial activity. This activity is primarily caused by an increase of reactive oxygen and nitrogen species (ROS and RNS, respectively), as well as the ability of NO to induce oxidative and nitrosative modifications in DNA, lipids and proteins, ultimately resulting in the inhibition or killing of targeted pathogens. In the context of skin infections, NO has demonstrated efficacy against pathogens such as *Staphylococcus aureus*, including methicillin-resistant strains (MRSA) [51]; *Trichophyton rubrum* [52] and *Candida albicans* [53].

NO plays a significant role in combating various skin infections, including cutaneous leishmaniasis caused by the protozoan parasite *Leishmania*. As a key mediator in the immune response, NO controls *Leishmania* infections through multiple mechanisms, acting as an antimicrobial effector while also regulating inflammatory cell recruitment to limit parasite proliferation and minimize host tissue damage [54,55]. Notably, NO-releasing chitosan NPs have demonstrated dose-dependent efficacy in inactivating *L. amazonensis* in infected mice, highlighting their therapeutic potential [54,56]. To overcome the intrinsic limitations of NO, such as its short half-life and high reactivity with oxygen radicals various delivery strategies have been developed for its effective application in medical and dermatological contexts.

## 4 Strategies for skin targeted NO delivery

Directly delivery of NO gas is limited by rapid diffusion and lack of site specificity. Instead, biomaterials use chemical conjugation of NO donors, which release NO under physiological conditions. The two main classes of NO donors are *N*-diazeniumdiolates (NONOates) and RSNOs [57]. NONOates are formed by reacting amines with high-pressure NO, generating R–N–N(O^−^)–NO groups that spontaneously release NO in aqueous environments [58]. RSNOs, on the other hand, are formed via nitrosation of thiol groups (–SH), producing S–NO bonds (exemplified by GSNO). Different NO donor chemistries offer different stability and release profiles. NONOates decompose via proton-driven mechanism; at neutral pH they generally release NO more slowly, whereas in mildly acidic environments such as the skin surface (≈pH 4.5–5.5) protonation accelerates decomposition and can produce an initial burst of NO (for short–half-life donors). They are relatively easy to synthesize on polymers but can suffer from short half-lives (minutes to hours) if not stabilized [8]. RSNOs generally have better stability at ambient conditions; they decompose to NO more slowly and their breakdown can be accelerated by triggers such as heat, light, or the presence of copper ions [59]. These small molecules NO donors are stable under suitable conditions but may experience triggered or premature decomposition under specific environments (e.g., acidic pH, light, heat or metal ions), leading to burst and undesirable NO release​. To address these issues, biomaterial-based approaches focus on improving donor stability and controlling the release kinetics [60,61]. Two general strategies are typically used for incorporating NO into biomaterials: (a) chemical incorporation via NO-donor molecules that are bound to or within the material, and (b) physical encapsulation or adsorption of NO-releasing compounds or NO-loaded reservoirs in the material matrix. In chemical incorporation, NO donors are covalently grafted onto a polymer backbone. Covalent bonding prevents the donor from leaching out and often slows the NO release because the donor is held within the polymer network [62]. In physical encapsulation or adsorption, NO donors are physically entrapped or adsorbed within a biopolymeric matrix. In this approach, a pre-formed NO-releasing compound (such as GSNO or a NO-loaded nanoparticle) is mixed with the biomaterial without forming covalent bonds. This strategy often slows down NO release due to the increased viscosity of the medium, while also protecting the donor from premature degradation [56,[63], [64], [65], [66]].

Materials containing amine or thiol functionalities (*e.g*., amine groups or cysteine residues) can be chemically modified to carry NO donors such as RSNOs, NONOates and nitrites [67]. For example, Davari et al. developed a nitrosated gelatin sponge, demonstrating sustained NO release over several days [68]. Following nitrosation, the resulting *S*-nitrosated polymer acted as a secondary NO reservoir, releasing NO indirectly through transnitrosation and limited homolytic cleavage, thereby providing sustained antibacterial activity when applied as a wound dressing. In summary, both chemical conjugation and physical encapsulation strategies have shown promising results in NO-releasing skin therapeutics, their effectiveness depends on controlling release kinetics, minimizing off-target toxicity and structural stability. These limitations have driven the development of next generation stimuli-responsive materials that can deliver NO with spatiotemporal control, as discussed in the following section.

## 5 Stimuli responsive NO-delivering biomaterials for skin therapy

Building on earlier NO-releasing dressings and hydrogels, stimuli-responsive platforms have been developed to release NO in response to exogenous cues (light, ultrasound, temperature) or endogenous wound signals (pH, reactive oxygen species, enzymes), thereby improving spatiotemporal control. A summary of representative stimuli-responsive NO-releasing biomaterial systems, including stimulus type, material platform, NO donor chemistry, experimental models and therapeutic outcomes, is provided in Table 1.

**Table 1:** Summary of representative stimuli-responsive NO-releasing biomaterials for antimicrobial skin therapy, in terms of stimulus type, material platform, NO donor chemistry, testing model and key therapeutic outcomes.

Stimulus type	Biomaterial platform	NO donor	Trigger mechanism	Testing model	Therapeutic outcome	Ref
Temperature	Pluronic F-127/chitosan hydrogel	GSNO	Thermo-induced gelation and NO diffusion	*In vitro*: Vero cell viability; *P. aeruginosa* antibacterial assays)	Sustained NO release, antibacterial activity (*P. aeruginosa*), low cytotoxicity	[69]
Ultrasound + Light	Photo-mediated ultrasound system	Endogenous NO pathways	Photoacoustic cavitation, mechanotransduction, endothelial dysfunction at higher energy	*In vitro*: RF/6A chorioretinal endothelial cells in a PDMS vessel model	Low-energy ultrasound or laser alone increased NO and PGI2, whereas PUT reduced the level of increase in NO and PGI2 compared with ultrasound-only and laser-only treatments	[70]
Light (visible, ∼420 nm)	β-cyclodextrin nanocarriers	NOPD1 (photodonor)	Photoactivated stepwise NO release from NOPD	*In vitro*: MRSA and *Acinetobacter baumannii* planktonic antibacterial assays under light activation	Light-dependent antibacterial activity against MRSA and *A. baumannii*	[72]
Light (NIR, 660 nm)	Ce6-grafted oxidized hyaluronic acid hydrogel loaded with l-arginine	l-arginine	Ce6-generated ROS oxidizes l-arginine to release NO	In vitro: HUVECs and HDPCs. *in vivo*: Dihydrotestosterone (DHT)-induced androgenetic alopecia in female C57BL/6 mice	Reduced IL-6/TNF-α, improved angiogenesis, improved follicular microenvironment and promoted hair regrowth	[73]
Hybrid (pH-responsive + NIR-assisted)	CuS@CaCO_3_ core-shell nanogenerator	Isosorbide dinitrate (ISDN)	Acid-triggered shell decomposition releases CuS and ISDN; NIR further improves NO release	*In vitro*: *E. coli* and *S. aureus* antibacterial/biofilm assays and HUVEC migration assays *In vivo:* infected diabetic wounds in female C57BL/6 mice	Strong antibacterial and antibiofilm activity, reduced inflammatory cytokines, enhanced angiogenesis, accelerated re-epithelialization, and improved diabetic wound closure	[74]
Hybrid (photothermal + NO)	Corrole/mPEG-SNO nanoparticle-loaded oxidized hyaluronic acid/carboxymethyl chitosan hydrogel	S-nitrosothiol-modified PEG (mPEG-SNO)	660 nm laser-triggered photothermal heating induces cleavage of S–N bonds and controlled NO release	*In vitro*: *E. coli* and MRSA planktonic, biofilm assays, L929 fibroblast migration assay, HUVEC tube-formation assay, and L929/HUVEC cytocompatibility tests *In vivo*: MRSA-infected diabetic wound model in female Kunming mice	Improved antibacterial and antibiofilm activity, reduced inflammation, promoted angiogenesis and accelerated diabetic wound healing	[60]
Hybrid (magnetic + photothermal + catalytic)	Magnetic nanoparticle–protein fiber coated gelatin methacrylate (GelMA) hydrogel	GSNO	Cu^2+^-mediated catalytic NO generation + NIR photothermal antibacterial effect	*In vitro*: *E. coli* and *S. aureus* antibacterial assays *In vivo*: infected wound model in BALB/c mice	Efficient antibacterial activity, reduced fibrosis, improved angiogenesis, and scarless wound healing	[75]
ROS-responsive	Poly(vinyl alcohol)–phenylboronic acid crosslinked hydrogel (TSPBA-based)	l-arginine	H_2_O_2_-triggered NO generation and and ROS-responsive hydrogel degradation	*In vitro*: ampicillin-resistant *E. coli* antibacterial assay *In vivo:* full-thickness infected wound model in Balb/c mice	Synergistic antibacterial activity, accelerated wound healing, enhanced collagen deposition and neovascularization	[76]
ROS-responsive	Supramolecular hyaluronic acid/l-arginine/Ce^3+^ hydroge	l-arginine	ROS-triggered in situ NO generation from l-arginine and Ce^3+^/Ce^4+^-mediated O_2_ generation	*In vitro*: L929 fibroblasts, HaCaT keratinocyte scratch assay, HUVEC tube-formation assay, RAW264.7 macrophage *In vivo*: skin wound model in diabetic male SD rats	Reduced oxidative stress and inflammation, improved angiogenesis, re-epithelialization, dermis regeneration and skin tensile strength	[77]
Multiple stimuli-responsive (pH/NIR/temperature/nanozyme)	Carboxymethyl chitosan/poly(N-isopropylacrylamide) cryogel loaded with MoS_2_–PDA–l-arginine nanozyme	l-arginine	NIR-triggered ROS generation induces NO release; pH-responsive bacterial capture; nanozyme-mediated ROS regulation; synergistic NO-assisted photodynamic/photothermal therapy	*In vitro*: MRSA and *E. coli* antibacterial assays, biofilm *In vivo:* MRSA-infected wound model in mice	Near-complete antibacterial activity (∼98–100%), effective biofilm eradication, improved angiogenesis and accelerated infected wound healing	[78]

### 5.1 Temperature/thermo-responsive materials

Thermo-responsive systems exploit lower critical solution temperature behavior to facilitate in situ gelation and local retention at skin temperature. Poloxamer-based hydrogels are attractive because of their injectability and biocompatibility, although their mechanical weakness and rapid erosion often require polymer blending. A representative example is the GSNO-loaded Pluronic F-127/chitosan hydrogel reported by Pelegrino et al., [69]. which showed sustained NO release for ∼24 h, antibacterial activity against *P. aeruginosa* and low mammalian cytotoxicity.

#### 5.1.1 Ultrasound-responsive NO release

Ultrasound offers a non-invasive means to trigger localized NO delivery by increasing skin permeability and inducing carrier disruption or donor decomposition. In this context, Karthikesh et al. [70] showed that photo-mediated ultrasound could increase NO and prostacyclin release, likely through mechanotransduction and photobiomodulation. Although this approach may be relevant to wound healing applications, translation remains limited by penetration depth, energy control and the challenge of achieving uniform activation *in vivo*.

#### 5.1.2 Light-triggered NO-release

Light-responsive NO delivery offers spatial and temporal control over NO release Photoresponsive NO donors, such as RSNOs and NONOates can be conjugated with nanoparticles or polymeric matrices that release NO upon exposure to visible or near-infrared (NIR) light [71]. Martins et al. [72] encapsulated a hydrophobic NO photodonor (NOPD1) within β-cyclodextrin branched polymer nanocarriers, improving NO bioavailability and showing antibacterial activity against MRSA and *Acinetobacter baumannii*.

A NIR-responsive hyaluronic acid/Ce6 hydrogel has also been reported for transdermal treatment of androgenetic alopecia, where light-generated ROS promoted l-arginine conversion to NO-related species and improved inflammation and hair regrowth *in vivo* [73]. Overall, light-sensitive NO-releasing systems provide highly tunable and non-invasive control over NO release, although, clinical translation will depend on improving penetration depth and reduced phototoxicity.

#### 5.1.3 Hybrid and multi-stimuli platforms

**Fig. 2:**
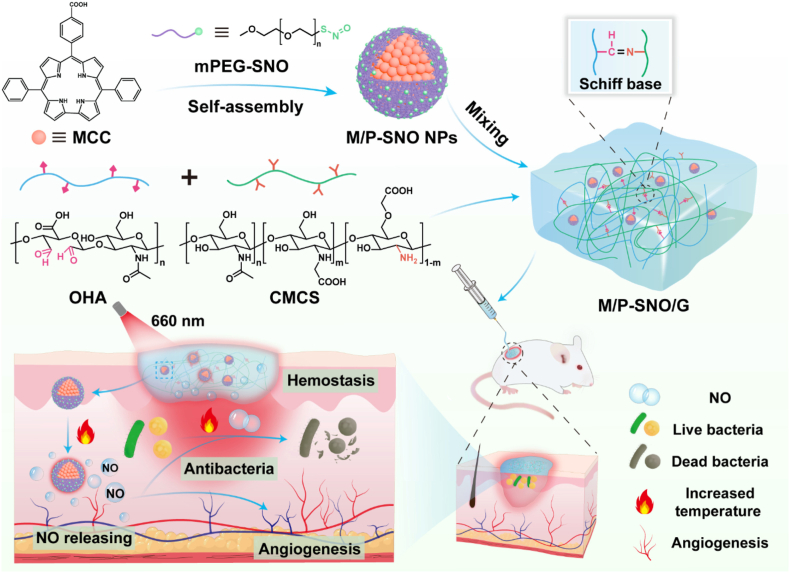
Schematic representation of the photothermal and NO-releasing nanocomposite hydrogel for diabetic wound healing, showing nanoparticle preparation, hydrogel formation, 660 nm-responsive NO release, antibacterial effects, hemostasis and angiogenesis. Reproduced from Yang et al., *Acta Biomaterialia* (2025) [60], with permission from Elsevier.

Hybrid and multi stimuli systems aim to combine environmental responsiveness with complementary functions such as photothermal therapy, catalytic NO generation or structural wound support. For example, Zhou et al. [74]. developed a pH-responsive core-shell nanogenerator that triggered NO release in acidic infected wounds and improved healing in diabetic models. Yang et al. [60] reported a photothermal/NO dual-responsive nanocomposite hydrogel (Fig. 2), which combines laser-triggered photothermal effects with controlled NO release to achieve antibacterial activity, hemostasis and improved angiogenesis in diabetic wound healing. He et al. [75] further combined catalytic NO generation with photothermal antibacterial effects in a magnetic nanocomposite dressing, although fabrication complexity and potential metal related toxicity remain translational concerns.

### 5.2 Endogenous stimulus-responsive systems

Endogenous wound-associated signals, including acidic pH, elevated ROS and enzyme upregulation, provide biologically relevant triggers for localized NO release. Because these cues arise directly from infection and inflammation, they may better match NO delivery to the wound microenvironment.

#### 5.2.1 pH-responsive systems

The pH of chronic and infected wounds generally ranges 5.5–6.5,allowing pH-responsive materials to remain relatively stable at physiological pH but activate under disease-relevant acidity. A representative example is the CuS@CaCO_3_ core-shell nanogenerator loaded with isosorbide dinitrate, in which dissolution of the CaCO_3_ shell in acidic wounds triggered localized NO release, antibacterial activity and improved healing in diabetic models [74].

#### 5.2.2 ROS/redox-responsive systems

Elevated ROS levels in inflamed and infected tissue can be exploited as endogenous triggers for localized NO generation. Yu et al. [76] reported an injectable ROS-responsive l-arginine hydrogel in which H_2_O_2_ promoted both hydrogel degradation and NO generation, reducing MRSA burden and improving re-epithelialization and collagen deposition. Similarly, Chen et al. [77] developed a ROS-responsive hyaluronic acid hydrogel that acilitated NO/O_2_ co-generation under oxidative conditions, improving bacterial control and tissue regeneration in diabetic wounds. Together, these studies highlight the potential of ROS-responsive systems to couple antimicrobial activity with wound repair. Notably, most ROS-responsive wound-healing systems employ low micromolar H_2_O_2_ concentrations (≈100–200 μM) [76,77], which are sufficient to trigger therapeutic responses while remaining within physiologically relevant and non-cytotoxic ranges.

#### 5.2.3 Enzyme-responsive nanocarriers for NO release

Enzyme-responsive approaches remain less explored, but they offer another route for site-selective NO generation in infected wounds. Yang et al. [78] developed a bacteria-responsive hydrogel containing a multifunctional nanozyme (MoS_2_–CuO_2_) that catalyzed NO release from l-arginine–chitosan complexes under acidic conditions, improving antimicrobial activity and tissue repair. Further mechanistic and translational studies are still needed.

## 6 Future outlook and translational challenges

Most stimuli-responsive NO releasing biomaterials for skin therapy remain at the preclinical stage, with evidence primarily derived from *in vitro* antimicrobial assays and small animal wound models (e.g., murine or rat diabetic wound models). These studies demonstrate proof-of-concept efficacy, clinical evidence in humans is still limited. The responsive materials for the delivery of NO are promising due to their ability to improve stability and biocompatibility, as well as spatiotemporally controlled NO release. Such systems can be tuned to respond to specific physiological triggers either intrinsic (such as pH, redox potential, enzymatic activity), or external triggers (such as light or ultrasound). However, despite the good pre-clinical results, the clinical translation of NO-releasing materials remains challenging. Key challenges include limited skin permeability due to the stratum corneum, restricted penetration depth of external triggers (such as light), and precise control of NO release kinetics *in vivo*. In addition to these challenges, regulatory considerations will play an important role in clinical translation, particularly for combination products that combine biomaterials with NO-donating compounds, which may require classification as drug device products. Long term safety of such responsive biomaterials remains insufficiently understood, in terms of potential cytotoxicity, off-target nitrosative stress and the fate of degradation by-products following repeated or chronic application. Precise control over NO dose and release kinetics is also essential, as both insufficient and excessive NO levels can impair healing or induce tissue damage.

External triggers such as light or ultrasound, have a challenge of penetration depth and uniform activation (or delivery) of source. Achieving precise control over NO dose and release kinetics in order to maximize therapeutic outcome remains an issue as well. This issue could be overcome with iterative optimization of materials properties and creating layer-by-layer hybrid systems by controlling their size-dependent surface chemistry. Thirdly, the requirement of real-time monitoring and feedback system is highly important especially for device free delivery systems such as enzyme- or redox-sensitive releasing systems. Combination with wearable biosensors may allow real-time feedback-controlled NO delivery. Finally, the issues of materials synthesis and characterization standardization and scalability, as well as their biodistribution and clearance and excretion pathways are important in translating these responsive systems to solve real-world clinical problems. To date, clinical studies evaluating stimuli-responsive NO biomaterials in dermatological applications remain scarce. Future work should prioritize large animal studies, safety evaluation *in vivo* and early phase human trials to bridge the gap between laboratory work and clinical potential.

## CRediT authorship contribution statement

**Kelli C. Freitas Mariano:** Conceptualization, Data curation, Formal analysis, Funding acquisition, Investigation, Methodology, Project administration, Resources, Validation, Visualization, Writing – original draft, Writing – review & editing. **Joana C. Pieretti:** Data curation, Formal analysis, Funding acquisition, Investigation, Methodology, Project administration, Resources, Validation, Visualization, Writing – original draft, Writing – review & editing. **Renan S. Nunes:** Formal analysis, Funding acquisition, Investigation, Methodology, Project administration, Resources, Validation, Visualization, Writing – original draft, Writing – review & editing. **Roberta A. dos Reis:** Data curation, Formal analysis, Funding acquisition, Investigation, Methodology, Project administration, Resources, Validation, Visualization, Writing – original draft, Writing – review & editing. **Victor D.P. Cinel:** Data curation, Formal analysis, Funding acquisition, Investigation, Methodology, Project administration, Resources, Validation, Visualization, Writing – review & editing. **Cecília B. Aragão:** Data curation, Formal analysis, Funding acquisition, Investigation, Methodology, Project administration, Resources, Software, Supervision, Validation, Visualization, Writing – original draft, Writing – review & editing. **Morgana Halfeld:** Conceptualization, Data curation, Formal analysis, Funding acquisition, Investigation, Methodology, Project administration, Resources, Software, Validation, Visualization, Writing – review & editing. **Tanveer A. Tabish:** Formal analysis, Funding acquisition, Investigation, Validation, Visualization, Writing – review & editing. **Amedea B. Seabra:** Conceptualization, Data curation, Formal analysis, Funding acquisition, Investigation, Methodology, Project administration, Resources, Software, Supervision, Validation, Visualization, Writing – original draft, Writing – review & editing.

## Declaration of competing interest

Tanveer Tabish is an inventor on a UK patent application entitle ‘NO generating material’ (filed by Oxford University Innovation) relating to nitric oxide (NO) generating stent coatings. The other authors declare no conflict of interest.

## Data Availability

No data was used for the research described in the article.
